# Seeking medical services among rural empty-nest elderly in China: a qualitative study

**DOI:** 10.1186/s12877-022-02911-0

**Published:** 2022-03-14

**Authors:** Yijin Wu, Quan Zhang, Yan Huang, Sihang Qiu

**Affiliations:** 1 School of Translation Studies, Qufu Normal University, 80 Yantai north road, Rizhao, 276825 People’s Republic of China; 2 School of International Affairs and Public administration, Ocean University of China, 238 Songling Road,, 266100 Qingdao, People’s Republic of China; 3 Centre for Quality of Life and Public Policy, Shandong University, 72 Binhai Road, Qingdao, 266237 People’s Republic of China

**Keywords:** Empty-nest elderly, Rural areas, China, Seeking medical services

## Abstract

**Background:**

The number of empty-nest elderly in China is rapidly increasing. Empty-nest elderly could not receive adequate daily care, economic support and spiritual consolation from their children. Rural empty-nest elderly are facing more serious health challenges than those in urban areas.

**Objective:**

This study aimed to understand the experiences of rural empty-nest elderly in seeking medical services in China.

**Methods:**

The method of inductive content analysis was used to collect and analyze data. Data were collected by in-depth interviews. A total number of 16 participants were involved in this study. A semi-structured interview guideline, which was discussed in depth and agreed upon by all researchers, was used to encourage participants to talk about their experiences in seeking medical services.

**Results:**

Rural empty-nest elderly is facing a great challenge in seeking medical services in China. There are some barriers for rural nest elderly to get access to healthcare services, such as low-income status, high expenditure of medical treatment and inadequate health insurance coverage. Due to the absence of the companionship of their adult children, empty-nest elderly have to rely on their neighbors and relatives to seek medical services.

**Conclusions:**

Rural empty-nest elderly have great difficulty in seeking medical services in China. More efforts should be made to get medical services more accessible to rural empty-nest elderly.

## Background

In China, the “empty-nest elderly” are the elderly people who do not have children, or who do not live together with their children, and thus, they live alone or with their spouses [1, 2]. With the declining birth rate and the trend of young people to live independently after marriage, the number of empty-nest elderly is rapidly increasing [3]. In 2015, the empty-nest elderly accounted for 51.9% of the elderly population [4], and the proportion has been predicted to reach 90% by 2030 [5].

Empty-nest transitions pose physical and mental health challenges to the elderly people [6–9], which is especially significant among empty-nest elderly in China. Compared with empty-nest elderly in other countries, Chinese empty-nest elderly could experience more emotional challenges due to their strong emotional dependence on their children and strong desire for intergenerational togetherness and co-residence [10, 11]. Accordingly, Chinese empty-nest elderly are more vulnerable to anxiety, loneliness, depressive symptoms and other mental illness [12–14]. Also, empty-nests have a significantly adverse influence on the elders’ physical health. Specifically, the prevalence of hypertension, diabetes, cerebrovascular disease, heart disease and other chronic or serious illness among empty-nest elderly is relatively higher compared with non-empty-nest elderly [15–18]. For example, a sample survey in Beijing showed that the prevalence of chronic conditions among empty-nest elderly was up to 84.55% [19]. Another survey in Shanghai found that the prevalence of chronic conditions among empty-nest elderly was as high as 90.5% [20].

Rural empty-nest elderly, who account for more than half of empty-nest elderly in China, face more serious health challenges than those in urban areas. Medical facilities and services in rural areas are weaker compared with that in urban areas. At present, medical facilities in most China’s rural areas are only able to deal with minor illnesses and do not have the capacity to treat serious illnesses [21]. In this sense, it is hard for empty-nest elderly in rural areas to get access to high -quality medical services [22]. According to *China Statistical Yearbook* in 2018, the number of doctors and registered nurses per 1000 people in rural areas was only 1.82 and 1.80 respectively, far lower than that of 10.91 and 5.08 in urban areas [23]. In addition, highly-educated medical personnel are in severe shortage in rural areas [24]. Accordingly, seeking medical services has become one of the most challenging problems faced by rural empty-nest elderly in China [25].

A very small number of studies have been conducted to investigate the phenomenon of seeking medical services among rural empty-nest elderly in China [26, 27]. Most of the existing studies are quantitative ones, and little qualitative studies have been conducted to explore the experiences of rural empty-nest elderly in getting access to medical care services. The qualitative research on the experiences of rural empty-nest elderly in seeking medical services can not only provide us with a deeper understanding of this social problem, but also demonstrate a comprehensive dimension of the availability and accessibility of healthcare servcies for rural empty-nest elderly in China. Using the method of inductive content analysis, this study aimed to examine the experiences of rural empty-nest elderly in seeking medical services in China.

## Methods

### Study design

In this study, inductive content analysis was used to collect and analyze data [28]. This method could provide a deep understanding of participant’s experiences and perspectives [29, 30]. It also can help the researchers to create concepts, categories, and themes, which can be extended to develop models, conceptual structures and theory [31]. The research process of this study consists of three phases: preparation, organization, and reporting of results [32]. In preparation phases, a total number of 16 participants were involved in this study. Face-to-face interviews were conducted to explore the experiences of rural empty-nest elderly in seeking medical services. In organization phases, the collected data was analyzed, categorized and themes were extracted [33]. Finally, the results were presented according to the Consolidated Criteria for Reporting Qualitative Research (CO-REQ) in the reporting phase [34].

### Participant recruitment

This study was conducted at two villages in Shandong Provinces, China, from June, 2020 to November, 2020. There are a number of empty-nest elderly living in the selected two villages, thus we could recruit a sufficient number of participants [35, 36]. In this sense, we selected two villages following the principle of “convenience” [37, 38]. Specifically, the two authors of this study lived near the two selected villages, which facilitates them as interviewers to get access to the participants. Moreover, since most of rural elderly in China could only speak the dialect rather than Mandarin, the two interviewers of this study can use the local dialect to communicate smoothly with participants. In addition, it should be noted that we selected two villages which near the two author’s villages rather than the villages where the two interviewers live, which could avoid the fallacy of prescience caused by the over-familiarity of interviewees with the participants [39, 40].

In this study, a purposive sampling method was employed to collect data. The inclusion criteria for participation enrollment were as follows: (1) the age of the participants should be 60 or older, and thus those younger than 60 were excluded from the survey; (2) the participants did not live with their children; we excluded those who occasionally lived with their children; (3) the participants should not suffer from serious deafness, severe Alzheimer’s disease and other serious mental illness, which could ensure that they can understand our questions clearly and respond to them efficiently.

### Data collection

In this study, semi-structured interviews lasted 15 to 40 min and were performed by two trained researchers. Before interviews, researchers accounted for the purpose of this study to all participants in a clear and understandable way. A semi-structured interview guideline (Table 1), which was discussed in depth and agreed upon by all researchers, was used to encourage participants to talk about their experiences in seeking medical services. Interview questions began with the participant’s experience of empty-nest life, then expanded to their experiences and perceptions of their illness, and finally focused on their experiences in seeking medical services. In addition, their expectations for improved access to medical services were also included. The audio recordings of the interviews were transcribed by YH and SQ, and then checked by QZ to ensure the accuracy of data transcription. Data were collected and analyzed until theme saturation occurred [41] after 14 interviews. After that, we conducted two more interviews to verified data saturation, and no new information had emerged. A total number of 16 participants were involved in this study.

**Table 1:** Interview topic guide

***Pre-designed questions***	***Prompts questions***
**The experience of empty-nest life**	
•What are your main recreational activities?	
•Do you live with your children?	•If not, what are the reasons for not being able to live with your children?
**The experience and perception of their illness**	
•What is the most serious illness you’ve had in recent years?	•How do you feel when you are sick?
•What was the most difficult thing you had when you were sick?	•How do you deal with these difficulties?
**The experience of seeking medical services**	
•What kind of treatment will you take when you are ill? (Simple treatment, intermittent treatment, regular treatment, or no treatment.)	•Can you tell me about what has influenced your decision?
•Where do you go to seek for treatment when you are sick? (village clinic, township central clinic, city hospital)	•What has influenced your choice?
•Is it convenient for you to visit the doctor when you are sick?	•If not, what are the specific reasons?
•Who usually accompany you to visit the doctor? (by yourself, with your relatives or family members)	•How do they provide help? •What is your feeling about their help?
**Expectations for improved access to medical treatment and service**	
•What do you think the government should do to deal with the difficulty of empty-nest elderly in seeking medical services?	

### Data analysis

The method of inductive content analysis was used in this study. Inductive content analysis is a qualitative method for the systematic description and interpretation of the collected data [42]. In this study, we follow the scientific analysis process of content analysis proposed by Elo and Kyngäs, which consist of three phases [42]. In open coding phase, two researchers read through the transcribed data and wrote down headings about the participants’ experience independently [43]. In category creation phase, the list of categories were grouped under higher order headings [44, 45]. In the abstraction phase, subcategories with content similarities were grouped as main categories [46]. To increase the trustworthiness of this study, two researchers coded all interviews independently. In cases where the two researchers could not agree on date reduction or abstraction, a third researcher was invited to review the codes. Coding disagreements were discussed until consensus was reached and the issue resolved [47]. In addition, during the coding process, codes were expanded and changed to ensure codes were extremely exhaustive [48]. Furthermore, feedback loops were used to ensure the rigor of this research [28].

## Results

The demographic characteristics of participants were showed in Table 2. In this study, 5 themes and 15 subthemes that were not mutually exclusive were identified.

**Table 2:** Participant’s characteristics

***Variables***	***N (16)***	***Percentage (%)***
**Gender**		
Male	8	50.00
Female	8	50.00
**Age ranges**		
60–65	2	12.50
66–70 70–80	6	37.50
71–75	3	18.75
76–80	3	18.75
80–100	2	12.50
**Number of children**		
1	2	12.50
2	7	43.75
3	5	31.25
4	2	12.50
**Living with spouse**		
Yes	7	43.75
No	9	56.25
**Health condition**		
Healthy without illness	3	18.75
With minor illness	2	12.50
With mild chronic illness	4	25.00
With serious chronic illness	7	43.75

### Theme 1: Barriers to seeking medical services

Although the government has taken a number of measures to help rural empty-nest elderly get access to healthcare services, there are still some barriers for them to get access to healthcare services.

#### Not having insufficient funds to pay for high medical costs

Almost all the participants said that they did not have enough money to pay for their medical costs. As a participant stated, “The income I receive is so low that I do not have enough money to pay my medical bills [participant 4]”. Participant 8 also conveyed similar worry, “I receive a subsidy of RMB1000 from the government every year, which is far from enough to cover the expensive medical treatments.” Some participants even gave up medical treatment because of their low-income status. “I have suffered many types of chronic conditions. I only get a small number of subsidies from the government each year. I will not have money to support my basic needs after paying my medical bills. Thus, although I suffered from an illness, I would not like to go to visit the doctor [participant 12]” In addition to the low-income status, some participants reported that the high cost of health care deters them from seeking medical services. “I would not like to see the doctor when I am ill, because it costs a lot of money. I just go to the pharmacy to get some medicine [participant 4]”. Another participant expressed the similar opinion, “I just take medicine to control my disease. I don’t want to be hospitalized because it will cost a lot of money. [participant 6]”. Furthermore, some participants claimed that the inadequate health insurance coverage hindered their access to medical services. Not all rural residents were coverd by the insurance system. According to participant 4, “I haven’t joined the New Rural Cooperative Medical Scheme (NRCMC) of our country, because I don’t have enough money to pay for the insurance.”

#### Lack of high-quality medical services in rural areas

Most participants argued that it is very hard for them to get access to high-quality medical services due to the lack of skilled healthcare professionals in the village clinic. As Participant 8 stated, “Due to the lack of highly skilled medical professionals, only minor illnesses could treated in the village clinic. If I have a serious illness, I can only call my son back and ask him to take me to the city hospital.” Another participant expressed his views on the insufficient supply of important medications “I need to take medications to treat the coronary heart disease I suffered. However, I could not get those medications in the village clinic. Therefore, I had to turn to my daughther for help. She would buy medicines online and then mail them to me [Participant 5]. Moreover, the lack of medical devices prevent participants from getting medical care in the villiage clinic. As participant 13 stated “Due to the lack of medical devices, many illnesses cannot be diagnosed and treated in the village clinic. I have to go to the city hospital for medical treatment, but it is too far. I have to travel 20 miles to got to the city hospital”.

### Theme 2: Emotional responses to their illness

Most of the recruited participants had suffered at least one chronic illness, and most of them had held negative feelings towards their illness. They stated that the illness made them feel psychological distress and inconvenienced.

#### Feeling psychological distress

Almost all participants mentioned that their chronic illness caused them unpleasant feelings. One participant mentioned that, “It is a bad thing to suffer from illness, and no one feels good when they are sick. I feel really bad about my illness” [Participant 3]. Another participant expressed similar feelings, “The skin disease I suffered embarrasses me. When the skin disease attacks, I feel upset[Participant 1]”. In addition to the unpleasant feelings, many partcipants bore great psychological pain, just as participant 13 stated “When arthritis sets in, my leg hurts so much that I even cannot walk. I am at my wit’s end! It brings me too much psychological pain”. Participant 10 also complained, “I suffered a lot from my disease. I feel extremely uncomfortable. It brings me too much psychological pain”. Furthermore, some participants showed the feeling of being scared because some illnesses can happen suddenly and even threaten their lives without timely treatment. According to participant 3, “I have been suffering from high blood pressure. Once upon a time, I fainted in front of my house. When I woke up, my neighbors told me that they had dialed the emergency call. I was lucky enough to be saved by my neighbors. If I faint again and no one sees me, what will I do?” Participant 4 also expressed the feeling of fear, “The year before last, I had a heart attack. I was moved to hospital quickly. I lived in the hospital for half a month ... If I was not sent to the hospital in time, you won’t see me now. I’m really scared when I think of this matter.”

#### Feeling inconvenienced by the loss of body function

Participants stated that the chronic illness suffered reduced or damaged their bodily functions, which adversely affected their daily activities and made them feel inconveniced. Some particpiants complained that chronic illnesses prevented them from taking simple exercises. As participant 4 said, “I could not take part in square dancing because I suffered heart disease and high blood pressure*.*” Another participant also stated, “When the arthritis set in on my leg, I could barely walk. In that situation, it is difficult for me to go outdoors, let alone travelling (sighed) [participant 12]”. Besides, participants who have suffered serious injuries would have problems with basic activities of daily living. Participant 2 made it clear that, “My knee was injured in 1958. I cannot stand up when I sit for a long time. I have to move my knee first and then slowly stand up… As a result, I cannot walk long distances at all and even cannot be able to visit the village clinic when I was ill. It’s too much hassle.” Participant 7 was in a similar situation, “My leg hurt the year before last, and my bones broke. Now, I cannot work, I cannot farm, I cannot do any job…How I wish I could work (sighed)!” Hearing loss is also a serious problem faced by rural empty-nest elderly, which made them feel troubled in daily communication. Just as participant 9 said, “I am hearing impaired, and I cannot hear sound clearly unless the speaker’s voice is loud.”

### Theme 3: Perception of their illness

This theme describes the participant’s cognitive appraisal and personal understanding of their illness. This theme consists of three sub-themes, that is, dealing their illness with peace of mind, underestimating the severity of their disease and cure being no longer expected.

#### Dealing their illness with peace of mind

Some particpants argue that the illness suffered shoud not influence their emotions. According to participant 9 who suffers from backache and deafness, “We should deal with our illness with a natural mood, and there is no need to be upset.” Some participants regard their illnes**s** as a natural result of aging. As participant 6 said, “It cannot be avoided that one suffers from illness when he/she grows older…As we get older, illness finds us by itself”. Some participants in this study even show indifferent attitude to their illness. As participant 15 stated, “Illness is not the whole of my life. There are many things to be happy in my life. We should have a good mindset. When we grow old, we have to take everything in stride.”

#### Underestimating the severity of their illness

It has been found that participants tend to underestimate the severity of their illness. Although they knew they had an illness, they did not take it seriouly in that their activities of daily living were not negatively influenced. Participant 6 mentioned, “Although I suffer from heart disease and high blood pressure, they do not affect my daily life. I think that my health condition is generally good.” Besides, most participants suffered two or more chronic conditions and they seem to have gotten used to the diseases suffered and no longer regard them as a very serious problem. Another participant with coronary heart disease and high blood pressure needs to take medicine three times a day; however, she still thinks her illness is not serious. “The medicine I have taken could control my illness well. Thus, in my opinion, my illness is not serious. [participant 5]”. Participants exprienced a worse situation, but they still consider it not serious. As participant 3 stated,“I had fainted from high blood pressure and was taken to hospital by ambulance once a time. After that, my husband would give me a massage, when I feel uncomfortable. This could relieve the symptoms of high blood pressure. I think high blood pressure does not matter to me”.

#### Cure being no longer expected

Many participants often no longer expect their illness to be completely cured. Some participants in this study suffered serious or intractable diseases. Participants with such diseases often no longer expect their illness to be completely cured. One participant noted, “Neither my heart nor my kidneys are in good health. I know that my illness is very serious and cannot be cured. [participant 7]”. Some participants suffered intractable disease, they give up the hope of the complete cure for their disease. Another participant, who had traveled to many cities to treat his intractable disease, finally gave up the hope of being cured. “It is impossible to cure my disease completely, because the sequelae of this disease has been going on for years... When the skin capillaries are blocked, it is difficult to restore it. Now I give up the hope of being cured. I just take some medicine to alleviate it [participant 1]”.

### Theme 4: Showing ambivalent attitudes towards the absence of their children’s accompaniment

Most participants reported that they looked forward to their children’s companionship in the process of visiting the doctor, while they could understand the absence of their chidren’s companionship.

#### Looking forward to their children’s companionship

Due to their reduced mobility*,* most of participants face great challenges in geting to the hospital when they were ill. Thus, they hope that their children could accompany them to visit the doctor. According to the views of one participant, “Two years ago, my leg had broken, which resulted in limited mobility. As a result, it’s not convenient for me to visit the doctor when I was ill. How I wish my children could accompany me to visit the doctor. However, my sons were not at home, and I had no daughter. So, I have to ask my neighbor’s son to go with me to see the doctor. [participant 10]”. In addition, some participants hope that their sons or daugthers could care for them during their hospitalization. As a participant stated, “The year before last, I had a heart attack. I was admitted to hospital for half a month. I really missed my two sons at that time, they both worked outside. How I wish they were by my side and cared for me at that time [participant 4]”. A few participants hope that their children could provide them with immediate assistance when they are in an emergency situation. Participant 3 said, “I have suffered high blood pressure. Once a time, the illness got worse and I fainted. My neighbors had dialed the emergency call. I have only one son, and he went out to work. My wife has passed away. How I wish my son could stay with me when I am in an emergency situation.”

#### Understanding the absence of their children’s companionship

Although many participants hoped to get the companionship of their children at a healthcare visit, they showed fully understanding of the absence of their children’s companionship. Due to the long distance between particiapnts and their children, some participants stated that their children’s absence could be understandable. Participant 4 mentioned, “I do want to live with adult children, who could take care of me. But this is not reasonable.…Their workplace is far from my home. It is not convenient for them to take care of me when I am ill”. In addition, some participants understood that their young adults are so burdened with their work that they could not have time to take care of their old parents. As a particpant stated “Yeah, I do want my children to stay with me and take care of me when I am sick. But they are so busy with their work that they can’t stay with me…I could understand (the reason for their absence) [participant 8]”. Several participants even expressed that they would not like to get their children’s accompaniment for fear of burdening them. “I do not expect my adult children to take care of me because it would add financial and time burden to them. They have not been married. They have to earn money for their marrige. [participant 6]”.

### Theme 5: External support for their accessibility of health care services

This theme focused mainly on external supporters who help participants get access to healthcare services. It has been identified that participants tend to seek medical services with the help of their neighbors, relatives and village doctors.

Without their children’s accompaniment and being limited mobility, many participants have to ask for their neighbors to take care of them and accompany them to seek medical services. “When I suffered from illness, my neighbors would drove me to see the doctor. Without their help, I cannot live till now. [participant 7]”. Indeed, a distant relative is not as good as a near neighbor, which could be strengthened by the 9th participant’s statement “My relatives do not live in the same village as me. Thus, I tended to ask for my neighbors to accompany me to visit the doctors when I was ill.”

In addition**,** some participants got support from their relatives who lived in the same village or the neighboring village in the process of seeking medical services. Their relatives helped them a lot in seeking medical services. As participant 7 said, “When I suffered from illness, my elder sister’s son would accompany me to visit the doctor. I have no children. I have to ask for help from my nephew.” Similarly, several participants stated that they had the experience of seeking medical services accompanied by their relatives who live in the same village as the participants, just as participant 13 stated “On one hand, I cannot drive. On the other hand, I am illiterate. However, the hospital is far away from my village. I live in the same village as my young brother’s son, who can drive and has received a good education. I would ask for him to accompany me to seek healthcare services when I was ill”.

Moreover, some participants got help from the village doctors when they were ill, as participant 15 stated “There is a clinic in my village, which I could get easy access to. The village doctor could provide medical treatment for some minor illnesses”. Moreover, the village doctors could provide home-based medical services for empty-nest elderly with minor illness. According to one participant, “I can’t walk easily as others. The village doctor would come to my home to provide medical treatment when I suffered from minor illness such as having a cold. The village doctor helped me a lot in seeking medical services [participant 5]”.

## Discussion

In this study, we investigated the experiences of rural empty-nest elderly in seeking medical services in China. Five themes have been identified, that is, the barriers for them to seek medical services, emotional responses to their illness, the perception of their illness, their ambivalent attitudes towards the absence of their children’s accompaniment, and social support for seeking medical services.

It has been identified that they are some barriers for rural empty-nest elderly to seek medical services, which is in line with the findings of previous studies. The Household Registration System (HRS) has brought about a huge income and pension inequality between the urban and rural residents [49, 50]. Financial difficulty became the leading cause of the lack of access to medical services for empty-nest elderly [51]. High expenditure of medical treatment caused by medical reform leaves rural empty-nest elderly with limited access to medical services [52, 53]. Also, the inadequacy of national health insurance further posed great economic challenge for rural empty-nest elderly in seeking medical services [54]. In addition to rural empty-nest elderly, the unemployed and low-income residents living in both rural and urban areas also face difficulties in seeking medical services due to the inequality of China’s national pension and health insurance system [55, 56]. To address these problems, Chinese Government launched a reform named “equalization of basic public services” in 2017, aiming to increase the pension income of the vulnerable groups, and improve their health insurance system to deliver better quality care at lower cost [57]. However, this will require significant financial outlays. In this sense, China still has a long way to go in promoting availability of high-quality medical services for the vulnerable groups.

In addition, this study demonstrated that rural empty-nest elders hold negative emotions towards their illness. This result was consistent with findings from De Ridder et al. [58] and Bužgová et al. [59], who found that chronic conditions tended to cause the empty-nest elderly negative emotions such as grief and affective disorder. The elderly people are especially vulnerable to negative emotions [60]. In line with the previous study [61], rural empty-nest elderly showed emotional insecurity when they suffered from illness. Our research findings also indicate that, the emotional insecurity could due mainly to the absence of the companionship of their adult children, which was not touched on by previous studies. Influenced by Confucian culture, Chinese elderly people have a close emotional connection with their children, namely, they tend to have a high degree of emotional dependence on their children [62, 63]. Thus, children’s homeleaving in Chinese culture could be seen as a breakup of family ties, which may cause considerable anxiety for their own elderly parents [64, 65].

In the third theme, it has been found that many participants regarded their illness as a normal result of aging, which was in line with previous studies on older Asian people [66, 67]. Engagingly enough, some participants tended to underestimate the severity of their illness, which has not be mentioned in previous studies. This may be explained by comparative psychology theory [68]. Specifically, since most of empty-nest elderly suffered from two or more chronic conditions, they seem to have gotten used to the diseases suffered and no longer regard them as a very serious problem. In this sense, rural empty-nest elderly in China tend to underestimate the severity of their illness. As a result, they may miss the best time for the treatment of their illness, which would make their illness worse.

A new phenomenon was identified in the fourth theme, that is, rural empty-nest elderly held ambivalent attitudes towards the absence of their children’ accompaniment in seeking medical services. On one hand, rural empty-nest elderly looked forward to receiving their children’s help in seeking medical services. On the other hand, many participants showed full understanding of the absence of their children’s accompaniment in medical care seeking. This may be explained by *parental sacrifice*, that is, parents tend to give up their personal needs for the sake of developmental needs of their children [69]. This study also found that many rural empty-nest elderly would not like to trouble their children and thus hide the bad news about their health from their children, which is another example of *parental sacrifice* in China [70, 71]. In addition to health problems, most aging parents would prefer not to bother their adult children with their other personal problems. It has been argued that family-oriented collectivism contributes a lot to the development of parental sacrifice for their children in China [72].

Three types of social support of rural empty-nest elderly have been identified in the last theme, that is, support from the neighbors, the relatives and village doctors. This could counteract the absence of their children’s accompaniment in seeking medical services. Our results also found that rural empty-nest elderly in China tended to get help from village doctors when they suffer from minor illnesses. This has not been reported in previous studies. Since the social networks of rural empty-nest elderly consists mainly of blood relatives, neighbors and village doctors, they could reach out to a very small number of people when help is needed [73]. It is clear that the existing social network is not favorable for them to seek medical services. Thus, we need to consider how to improve the availability and accessibility of medical servcies for rural empty-nest elderly. It has been found that volunteer medical visit companions could be able to assist older adults in seeking medical help [74]. With the growth of voluntary associations in China, volunteers could be trained to accompany rural empty-nest elderly to their medical appointments.

Due to the limited access to medical services and being limited mobility, rural empty-nest elderly encounter great inconvenience when seeking medical services. In addition, our results also indicate that the absence of the accompaniment of their adult children further worsen this kind of inconvenience. Although some studies highlighted the benefits of medical visit companions [75, 76], it is hard for Chinese young adults to accompany their own elderly parents to medical visits. At present, most of young adults in rural areas leave their hometown for cities in order to find a good job [77]. As a result, they can hardly provide adequate care for their empty-nest elderly parents [78]. Thus, it is necessary for Chinese government to increase its investment in rural healthcare. Notablely, Chinese government has launched the “rural revitalization” program, which could make healthcare accessible to a wider empty-nest elderly people in rural areas.

### Strengths and limitations

To the best of our knowledge, this is the first qualitative study to explore how Chinese empty-nest elderly in rural areas get access to medical service. Although many efforts have been made to ensure the trustworthiness of this study, there are still several limitations to this study. As with all qualitative research, the findings in this study could not be generalized. In addition, in this study, only empty-nest elderly who live in Eastern China has been investigated. Medical facilities and services in Eastern China is significantly better than that in Western China [79]. In this sense, rural empty-nest elderly in Western China may face more challenges in seeking medical services [80]. Further research is needed to investigate rural empty-nest elderly, who live in Western China.. Also, further research could be expanded to investigate the village doctors’ perceptions of health care for rural empty-nest elderly and explore health officials’ perspectives on how to improve rural healthcare access in China.

### Implications for practice

It is clear that rural empty-nest elderly in China are facing great challenges in seeking medical services and thus more efforts should be made to get medical services more accessible to them. First and foremost, more and more general practitioners should be encouraged to work in rural areas in China, and more medical facilities with high-quality medical services should be established in the rural areas. Second, the Elder Watch Program could be put into practice in China, which calls for volunteers to keep in regular contact with rural empty-nest elderly and accompany them to visit the doctor when needed. Third, barriers for rural empty-nest elderly to seeking medical services, such as high expenditure of medical treatment and the inadequate health insurance coverage, should be addressed gradually. This involves a more complex project where the hospital business model and the health care system should be reformed.

## Conclusion

Due to the absence of the companionship of their adult children, rural empty-nest elderly have great difficulty in seeking medical services. Many empty-nest elderly have to rely on their neighbors and relatives to seek medical services. In addition, there are some barriers for rural empty-nest elderly to get access to healthcare services, such as the low-income status, high expenditure of medical treatment and the inadequate health insurance coverage. Thus, more efforts should be made to get medical services more accessible to rural empty-nest elderly in China.

## Data Availability

The original data will not be shared in order to protect participant confidentiality, however further information which does not compromise confidentiality data can be obtained from the corresponding author on a reasonable request.
